# Improved performances of lithium-ion batteries using intercalated a-Si–Ag thin film layers as electrodes

**DOI:** 10.1039/c8ra07904h

**Published:** 2018-12-11

**Authors:** Pan Wang, Ling Tong, Rongfei Wang, Anran Chen, Wenzhong Fang, Kun Yue, Tao Sun, Yu Yang

**Affiliations:** International Joint Research Center for Optoelectronic and Energy Materials, Yunnan University Kunming 650091 China; School of Materials Science and Engineering, Yunnan University Kunming 650091 China; School of Energy, Yunnan University Kunming 650091 China yuyang@ynu.edu.cn; School of Physics and Astronomy, Yunnan University Kunming 650091 China

## Abstract

The laminated construction of an a-Si–Ag thin film electrode is demonstrated, which allows stabilization of the cycling performance of a silicon thin film layer in a lithium-ion battery. A silver thin film plays a determining role in the lithium insertion/extraction process and is incorporated between amorphous Si thin film layers (a-Si/Ag/a-Si), which results in not only high and stable capability, but also the best rate performance compared to that of other electrodes. For the electrode of a-Si/Ag/a-Si, a critical thickness of the silver layer (30 nm) is found; in this case, it exhibits the highest capacity retention of 70% after 200 cycles at a current density of 65.2 μA cm^−2^ within the voltage range of 0.01–1.5 V. It is demonstrated that for the a-Si/Ag/a-Si (140/30/140 nm) electrode, enhanced capacity (∼59.1%) is derived from the buffer effect and excellent conductivity of silver layer. Silver interlayer may represent a universal platform for relieving stress in a silicon electrode. In addition, its excellent electrical conductivity will decrease the charge transfer resistance of Si electrode for lithium-ion batteries.

## Abstract 2

A novel silver interlayer is used to improve the electrochemical performance of the binder-free Si-based thin film anodes.

## Graphical abstract

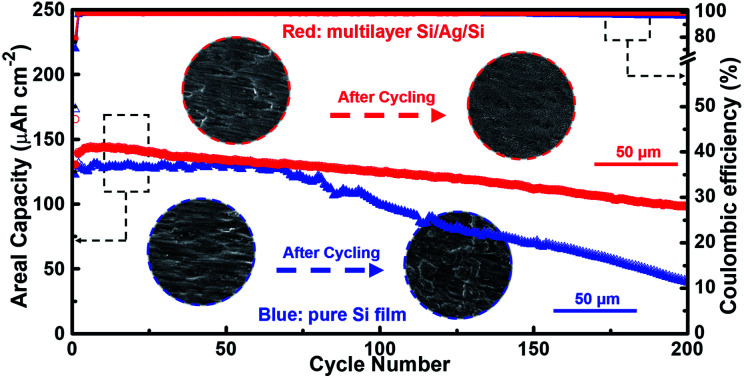

## 1. Introduction

Rechargeable lithium-ion batteries (LIBs) with higher power and energy density are widely used as the most effective energy storage devices, especially with the rapid development of innovative electronic products and electrical vehicles.^1^ However, the limited energy density, high power and stability are the biggest challenging issues for LIBs, rendering them difficult for use in practical vehicles.^2,3^ The commercial anode material for LIBs, namely, graphite cannot meet the above-mentioned needs in practical applications because of its low theoretical specific capacity (only 372 mA h g^−1^). A silicon thin film layer is one of the promising materials for the next-generation LIBs due to its high theoretical capacity (4200 mA h g^−1^) and low working potential (0–0.5 V *vs.* Li/Li^+^).^4–7^ However, a pristine silicon anode suffers from serious volume change (>300%) during lithium insertion/extraction processes, resulting in electrode pulverization and subsequent loss of electrical contact between the active material and the current collector, eventually leading to poor reversibility and rapid capacity fading.^8–10^ In addition, its low intrinsic electrical conductivity (10^−3^ S cm^−1^) impedes the electrochemical kinetics of the charge/discharge processes, which leads to moderate rate performance.^11,12^ The disadvantage of silicon thin film layers mentioned above can be addressed by reducing their thickness to a nanoscale size,^13^ compounding with metals (Ag,^12^ Cu,^14^ Mg,^3,15^ Ti,^16^*etc.*), or using modified collectors.^17–20^

Silicon-based multilayer electrodes, such as Si–C,^21–23^ Si–Ge,^24^ Si–Cr,^25^ Si–Co,^26^ or Si–Al^27^, can effectively relieve stress caused by volume changes as well as provide a short pathway for Li^+^ diffusion and electron transportation.^28^ In addition, they also display increased electronic conductivity compared to pure silicon.^29^ Therefore, they can retain a longer lifetime and exhibit more stable rate performances. In addition, silicon-based multilayer thin films directly grown on collectors contain no binders and are conductive, which decreases more than 10 wt% of the inactive materials, thus delivering increased specific capacity.^30,31^ The preparation processes of electrodes such as coating, drying and pressing are omitted as well. For example, Tobias Placke^29^ demonstrated that the multilayer Si/C/Si (70/50/70 nm) thin film electrode showed superior cycling performance after 150 cycles with capacity retention of 83 ± 6% compared to a pure Si thin film layer (74 ± 7%) because of the advantage of an additional interlayer that worked as a buffer layer and increased electronic conductivity.

Herein, a silver thin film layer is used to improve the performance of an a-Si thin film electrode. To study the influence of silver thin film layers on the electrochemical characteristics, Ag/a-Si, a-Si/Ag/a-Si, and a-Si/Ag electrodes are also fabricated by the magnetron sputtering technique. The multilayer system of a-Si/Ag/a-Si exhibits the most stable cycling performance after 150 cycles with the highest discharge areal capacity (111.7 μA h cm^−2^) and optimal capacity retention of 80% compared to other electrodes. In particular, the impact of the thickness of the silver layer based on the a-Si/Ag/a-Si structure is investigated. The proportion contributed by the silver thin film layer during cycling is further calculated to comprehend the constitution of the capacity. Furthermore, a major part of this study is assigned to investigate the electronic conductivity as well as mechanical stability during lithium insertion/extraction processes.

## 2. Experimental

### 2.1 Material preparation

Silicon and silver materials were grown on copper foil in a magnetron sputtering system (FJL560III-type). The base pressure in the sputtering chamber was approximately 3 × 10^−4^ Pa and Ar (99.999%) was invoked as the working gas. The Si thin films were deposited at a constant radio frequency power supply of 100 W. Ag thin films were deposited by direct current sputtering at an applied power of 40 W. In both cases, for Si and Ag thin films, the sputter processes were performed at room temperature with a working pressure of 1 Pa. The deposition rates calculated from the total film thickness were 4.67 nm min^−1^ for silicon and 30.0 nm min^−1^ for silver. To eliminate impurities present on the target surface, both silicon and silver targets were pre-sputtered for more than 10 min.

### 2.2 Material characterization and electrochemical measurement

The morphologies of the as-produced Si–Ag samples were characterized by field emission electron microscopy (FESEM, FEI Nova Nano SEM 450, Rigaku Corporation, Tokyo, Japan). Structural characterization was performed by X-ray diffraction (XRD, Rigaku TTRIII, Rigaku Corporation, Tokyo, Japan) operated at 35 kV and using Cu-Kα radiation (1.5406 Å) in the range of 5–90°. The micro-Raman spectra were recorded using backscattering geometry with Ar^+^ laser (514.5 nm) as the excitation source at room temperature. The X-ray photoelectron spectroscopy (XPS) analysis was performed on a K-Alpha^+^ instrument from Thermo fisher Scientific using monochromatic Al Kα radiation (72 W, 6 mA, 12 kV) and high energy electron flooding for charge compensation.

The electrochemical properties of the Si–Ag thin films directly deposited on Cu foil (14 mm diameter and 9 μm thickness) were tested using 2032-type half-cells in an argon-filled glove box. The assembled cells contained Si–Ag thin film as the working electrode, an electrolyte, a separator (Celgard 2325) and a lithium metal foil as the counter electrode. The electrolyte was 1 M LiPF_6_ in a mixture of ethylene carbonate (EC) and dimethyl carbonate (DMC) (1 : 1 in volume ratio).

Galvanostatic charge–discharge and rate performance tests were carried out on a battery testing system (Neware BTS3000) between 0.01 V and 1.5 V. An electrochemical workstation (chi 760e) was used to perform cyclic voltammetry (CV) and electrochemical impedance spectroscopy (EIS). CV was performed for the first 5 cycles in the potential range of 0.01–1.5 V (*vs.* Li/Li^+^) at a scan rate of 0.1 mV s^−1^ and EIS measurements were obtained in the frequency range from 100 kHz to 0.01 Hz after 5 cycles. The cycled samples were disassembled in the glove box and cleaned separately with acetone and ethanol and dried naturally.

## 3. Results and discussion

**Fig. 1:**
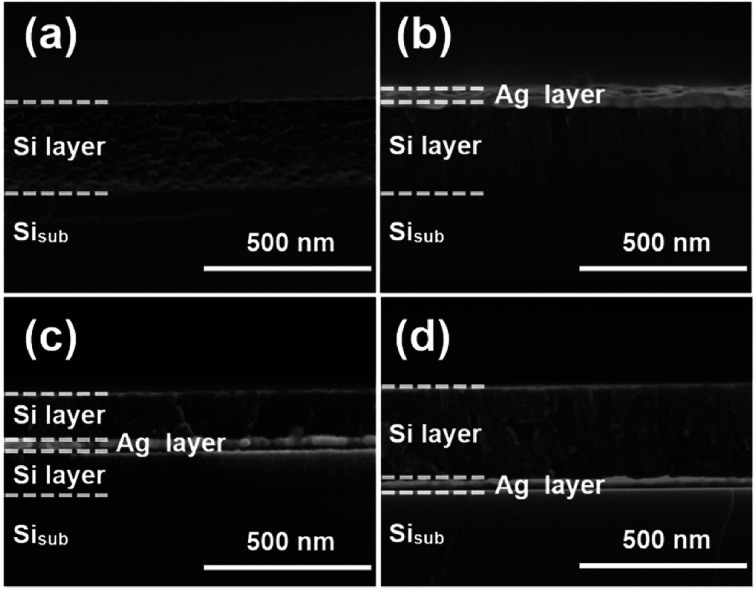
Cross-sectional SEM images of the Si–Ag film with different positions of the Ag thin layer: (a) Si (280 nm)/Si_sub_; (b) Ag (30 nm)/Si (280 nm)/Si_sub_; (c) Si (140 nm)/Ag (30 nm)/Si (140 nm)/Si_sub_, (d) Si (280 nm)/Ag (30 nm)/Si_sub_. “Si_sub_” stands for silicon substrate.

Fig. 1 shows the cross-sectional SEM images of a typical nanocomposite of four designed structures: pure silicon thin film, silver/silicon thin film, silicon/silver/silicon thin film, and silicon/silver thin film. To facilitate cutting, all samples were deposited on *n*-(100) single crystalline silicon substrates. The pure amorphous silicon layer was about 280 nm in size (Fig. 1(a)) and was compared with the modified samples. A silver thin film layer was deposited on top of the Si layer (denoted as Ag/Si/Si_sub_). About 30 nm Ag thin film was deposited on the surface of pure Si layer to protect the susceptible Si electrode/electrolyte interface, and the rigid structure could limit the volume expansion of silicon during the process of lithiation and delithiation (Fig. 1(b)). From Fig. 1(c), we can observe that it is a multilayer Si/Ag/Si/Si_sub_ system and the thicknesses were set to 140, 30, and 140 nm, respectively; due to this, the total thickness of the silicon layer remained constant and was used to study the effect of different positions of Ag thin film layer on the cycling performance. Fig. 1(d) shows that a 30 nm silver thin film was directly deposited before deposition of a 280 nm Si thin film, forming the Si/Ag/Si_sub_ system.

**Fig. 2:**
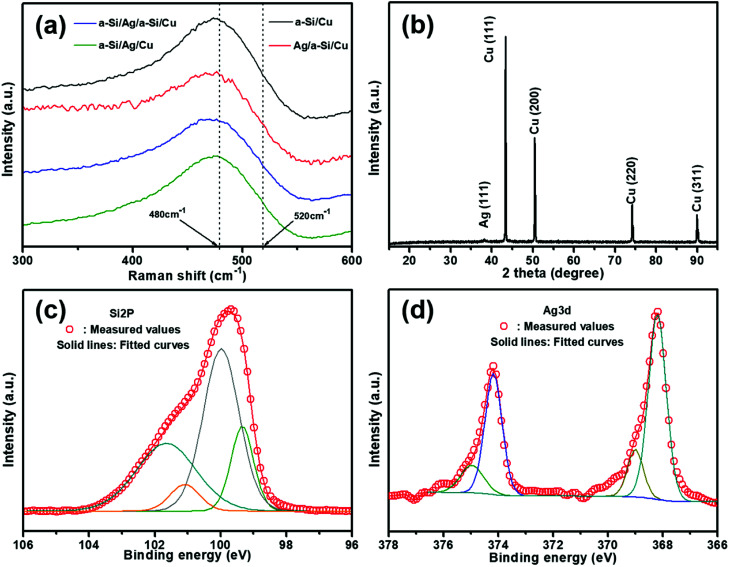
(a) Raman spectra of the pristine a-Si (280 nm)/Cu (9 μm), Ag (30 nm)/a-Si (280 nm)/Cu (9 μm), a-Si (140 nm)/Ag (30 nm)/a-Si (140 nm)/Cu (9 μm) and a-Si (280 nm)/Ag (30 nm)/Cu (9 μm) electrodes; (b) XRD pattern of a-Si/Ag/a-Si/Cu multilayer electrode; XPS spectra: (c) Si 2p spectra and (d) Ag 3d spectra of a-Si/Ag/a-Si multilayer film deposited by the sputtering method. The top silicon layer is removed by Ar ion etching.

Raman spectroscopy was used to investigate whether this structure is crystalline or amorphous or even a transition between these two phases.^18^ All samples exhibit strong vibration peaks, as demonstrated in the Raman spectra (Fig. 2(a)). A broad peak is observed and is centered around 480 cm^−1^ of Raman shift, which is generally assigned to amorphous silicon (a-Si). In addition, no indication of the crystalline silicon phase is shown since no apparent peak at 520 cm^−1^ is revealed.^18^ To further illustrate the existence of silver and silicon phases, the most representative electrode of a-Si/Ag/a-Si deposited on Cu foils is tested using XRD (Fig. 2(b)). There are four characteristic diffraction peaks at 43.3°, 50.4°, 74.3° and 89.9°, which can be assigned to the (1 1 1), (2 0 0), (2 2 0) and (3 1 1) planes, respectively, in the cubic phase of Cu. It is also seen that there is a weak diffraction peak at 38.1° related to the (1 1 1) plane of the cubic Ag phase. The diffraction peaks are ascribed to the Cu foil and the silver thin film layer, and no peak of Si appears, especially the typical peak for crystal Si at 28°. This also indicates that the Si thin film is amorphous.^5^Fig. 2(c) and (d) show the XPS spectra of an intercalated a-Si/Ag/a-Si multilayer film after removing the silicon top layer by Ar ion etching. The fitted Si 2p spectrum shows two evident peaks at 99.3 and 100.0 eV, which are assigned to the Si–Si band indexed to elemental Si.^32,33^ The characteristic peak at 101.7 eV refers to SiO, and the peak at 101.1 eV is ascribed to SiO_*x*_, which originate from the exposure of silicon to the ambient environment.^34–36^Fig. 2(d) shows the XPS spectrum of the Ag 3d region, in which the peaks at around 368.2 and 374.2 eV can be ascribed to metallic Ag.^37,38^ In addition to Ag–Ag bonding, peaks at 369.0 and 375.0 eV are also found, which actually indicate the existence of Si–Ag bonds in our samples; these results are consistent with those of other literatures.^39,40^

**Fig. 3:**
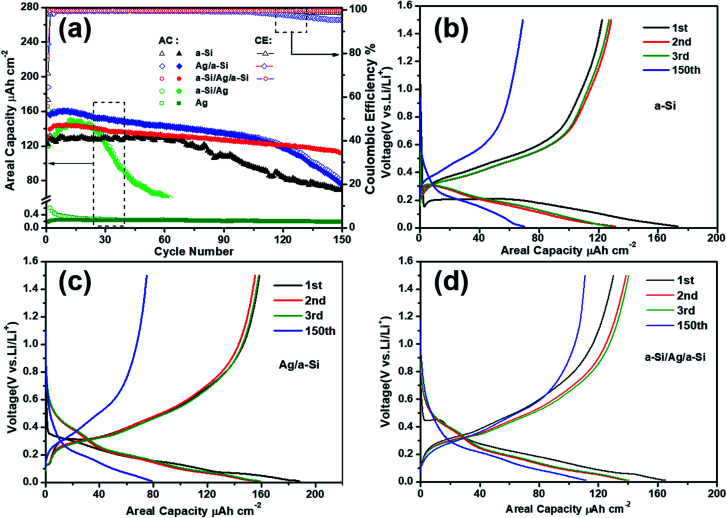
(a) The cycling performances of Ag, a-Si, Ag/a-Si, a-Si/Ag/a-Si and a-Si/Ag electrodes at a current density of 65.2 μA cm^−2^ with the voltage range of 0.01–1.5 V. The solid and hollow symbols correspond to the charge and discharge curves. “AC” and “CE” stand for “Areal Capacity” and “Coulombic Efficiency”, respectively. Charge/discharge voltage profiles of the 1st, 2nd, 3rd and 150th cycles for the pure a-Si (b), Ag/a-Si (c) and a-Si/Ag/a-Si (d) electrodes at a current density of 65.2 μA cm^−2^ with the voltage range of 0.01–1.5 V.

To demonstrate the effect of the position of the deposited silver thin film layer, the cycling performances of the Ag, a-Si, Ag/a-Si, a-Si/Ag/a-Si and a-Si/Ag electrodes were measured at the same current density of 65.2 μA cm^−2^ within the potential window from 0.01 to 1.5 V *versus* Li/Li^+^ by galvanostatic discharge and charge processes (Fig. 3(a) and Table 1). The areal capacity (μA h cm^−2^) is employed to evaluate the ability of Li-ions’ insertion/extraction in the anodes fabricated in this study, and it is defined as the total discharge/charge capacity (μA h) divided by the projected surface area (π × 7 mm × 7 mm ≈ 1.54 cm^2^) of the composite electrodes.^41^ It can be seen that the pure a-Si electrode delivers the first discharge capacity of 173.2 μA h cm^−2^ with an initial coulombic efficiency (CE) of 70.6% and displays the best cyclic stability for 70 cycles without a clear capacity loss. However, the discharge capacity degrades rapidly down to 70.2 μA h cm^−2^ after 150 cycles, indicating only 53.5% retention of the initial reversible capacity (Table 1). The poor cycling performance of the pure a-Si anode can be ascribed to the serious volume expansion during Li lithiation/delithiation processes.^28^ Compared to the pure Si electrode, the Ag/a-Si and a-Si/Ag/a-Si electrodes exhibit higher areal capacities and significantly retarded capacity fading processes. The Ag/a-Si electrode shows the highest first discharge capacity (188.2 μA h cm^−2^) and cycling CE (84.2%), but the capacity degrades rapidly after 115 cycles, which is similar to that observed for pure silicon electrode. During 1 to 150 cycles, for the a-Si/Ag/a-Si electrode, there is no observable sharp decline in capacity. It is clear that the a-Si/Ag/a-Si electrode possesses the most stable cycling performance with the highest discharge areal capacity (111.7 μA h cm^−2^) and optimal capacity retention of 80% after 150 cycles compared to other electrodes. Among all the materials, a-Si/Ag as the anode material shows the worst cycling performance, which indicates that the silver thin film deposited on the copper foil cannot increase the adhesion of silicon to the substrates such as Ti^22,23,42^ and Cr.^43–45^ The CE values after 150 cycles are 98.3%, 95.2%, 99.5% and 99.1% for the electrodes of a-Si, Ag/a-Si, a-Si/Ag/a-Si, and a-Si/Ag, respectively. Also, 30 nm-thick Ag layers as anodes were tested for comparison.

**Table 1:** Overview of the coulombic efficiencies and capacity retentions of different thin film electrodes

Electrodes	First cycle areal capacity/μA h cm^−2^	First cycle CE/%	Capacity retention in 150th cycle/%	150th cycle CE/%
Ag	1.96	9.3	30.9	96.3
a-Si	173.2	70.6	53.5	98.3
Ag/a-Si	188.2	84.2	50.2	95.2
a-Si/Ag/a-Si	165.4	78.8	80.0	99.5
a-Si/Ag	161.6	74	26.4	99.1

It is therefore suggested that the capacities of Ag/a-Si and a-Si/Ag/a-Si anodes are not the sum of the capacities of silver and silicon electrodes but a greater improvement thereof. Slight increase in the capacities for the a-Si and a-Si–Ag electrodes in the initial several cycles is linked to the gradual activation of the silicon host, which may result from the gradual infiltration of the electrolyte.^1,46^

Charge/discharge voltage profiles for pure a-Si, Ag/a-Si and a-Si/Ag/a-Si electrodes are depicted in Fig. 3(b), (c) and (d), respectively. It is clear that there are at least two voltage plateaus located between 0.1 and 0.6 V, indicating more than one step reactions due to lithiation and delithiation of Si; the potentials will be discussed below in detail. Regarding the discharge/charge profiles of the 150th cycle of the pure a-Si, Ag/a-Si and a-Si/Ag/a-Si electrodes, the patterns are still quite similar to the curves of the 3rd cycle. This indicates that the fundamental electrochemical process is stable even though some capacities are lost with increased cycling.^1^

**Fig. 4:**
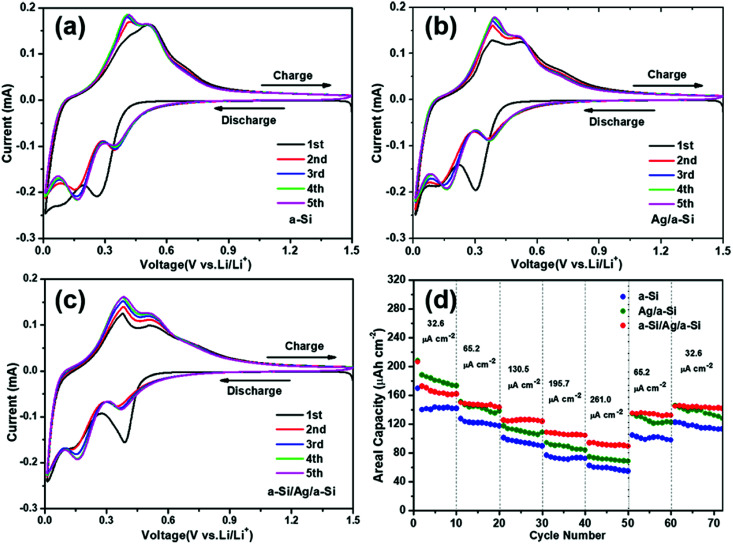
Cyclic voltammograms of the first five cycles of (a) pure a-Si, (b) Ag/a-Si, and (c) a-Si/Ag/a-Si thin film electrodes in the voltage window (*vs.* Li/Li^+^) of 0.01–1.5 V at a scan rate of 0.1 mV s^−1^; (d) the rate capabilities of the pure a-Si, Ag/a-Si, and a-Si/Ag/a-Si thin film electrodes.

In Fig. 4, the first five cycles of a CV measurement at a scan rate of 0.1 mV s^−1^ of pure a-Si thin film (a), Ag/a-Si (b), and a-Si/Ag/a-Si (c) electrodes are depicted. There are two cathodic peaks and two anodic peaks in all CV measurements, indicating the two-step lithiation as well as two-step delithiation processes, which is in good agreement with the charge/discharge voltage profiles (Fig. 3). The observed broad cathodic peaks in the potential region from 0.5 to 0.05 V *vs.* Li/Li^+^ in all CV measurements are ascribed to the formation of Li_*x*_Si alloys with different compositions.^8,29^ In the anodic scan, Li^+^ ion extraction from Si occurs from 0.2 to 0.6 V.^29,47,48^

In the case of the pure a-Si electrode, two cathodic peaks centered at 0.26 V and 0.09 V in the first cycle shift to 0.35 V and 0.16 V, respectively, in the second cycle (Fig. 4(a)). The potential shift of cathodic peaks indicates increased kinetic polarization and internal resistance.^17^ The Ag/a-Si electrode also shows a similar phenomenon. However, the same is not true in the case of a-Si/Ag/a-Si electrode, as shown in Fig. 4(c). Peaks related to the reaction between Ag and Li are not detected, which may be due to the relatively low contribution of Ag in Ag/a-Si and a-Si/Ag/a-Si electrodes.^49^ The intensities of both cathodic and anodic peaks gradually increase with increased cycling due to the activation processes at the electrodes.^11,50^

The formation of a solid electrolyte interface (SEI) layer should occur between 0.5 and 1 V, and it is usually indicated by a peak in the CV data around 0.8 V due to the dissociation of electrolyte.^51,52^ However, there is no such peak observed during the first discharge curve corresponding to the potential of SEI layer formation, which is consistent with the results of previous reports.^50,53,54^ Although SEI formation may occur, the capacity involved in SEI formation would be very small compared to the high discharge capacity we observed. Therefore, the initial irreversible capacity is not mainly caused by the formation of the SEI layer but possibly by the fracture of the Si film, and it becomes relatively stable in the subsequent cycles until larger areas of the structures collapse. Fig. 4(d) presents the discharge capacities at different current densities. It can be seen that the a-Si/Ag/a-Si electrode also exhibits a better high-rate performance than pure a-Si and Ag/a-Si electrodes. The a-Si/Ag/a-Si electrode delivers lower capacities than the Ag/a-Si electrode at a current density of 32.6 μA cm^−2^ in the first 10 cycles. However, perfect capacity retention is observed with increasing current densities due to excellent electron conductivity of the a-Si/Ag/a-Si electrode. The next 10 cycles were performed at 65.2, 130.5, 195.7 and 261.0 μA cm^−2^. In the case of the a-Si/Ag/a-Si electrode, the reversible capacities at different periods were 148.1, 124.0, 107.7 and 94.2 μA h cm^−2^, which were higher than those of pure a-Si and Ag/a-Si electrodes at the corresponding current densities (Table 2). When the current densities were switched back to 65.2 and 32.6 μA cm^−2^, the capacities of the a-Si/Ag/a-Si electrode were still higher than those of other electrodes, and the decline in capacities after 70 cycles was the most gradual as well. According to the above-mentioned results (Fig. 3 and 4), the a-Si/Ag/a-Si electrode not only showed impressive cycling performance, but also better high-rate capability.

**Table 2:** Rate performances of pure a-Si, Ag/a-Si and a-Si/Ag/a-Si electrodes at the first five current densities (32.6, 65.2, 130.5, 195.7, and 261.0 μA cm^−2^) depicted in Fig. 4(d)

Electrodes	Current density (μA cm^−2^)				
	32.6	65.2	130.5	195.7	261.0
a-Si	139.8	123.4	98.2	74.3	60.0
Ag/a-Si	187.9	146.4	113.6	91.2	73.1
a-Si/Ag/a-Si	172.3	148.1	124.0	107.7	94.2

Rate dependence is poor in all samples at different cycles, especially at the first five current densities (32.6, 65.2, 130.5, 195.7, and 261.0 μA cm^−2^). This is because the difference in current densities between two adjacent cycles is small. When the current density was switched from 261.0 μA cm^−2^ back to 65.2 μA cm^−2^, the capacities of all samples increased rapidly. For the a-Si/Ag/a-Si electrode, the capacity value increased from 89.2 to 134.8 μA h cm^−2^, corresponding to the 50th and 51st cycles. This huge capacity change was caused by the large change in current density. A similar phenomenon of the poor rate dependence can be found in previous research as well.^11,55–57^

**Fig. 5:**
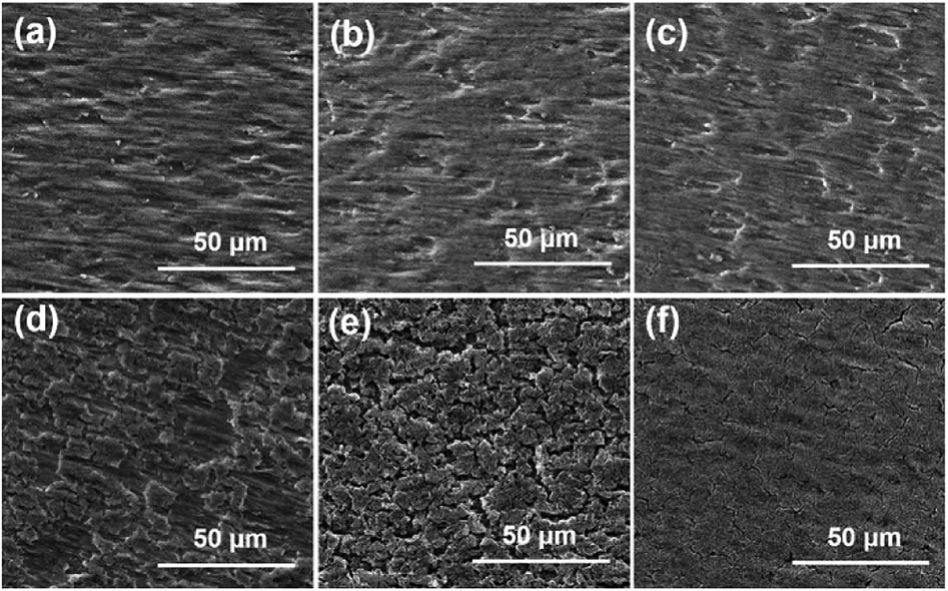
The SEM surface morphologies of pure silicon thin film layer (a and d), Ag/a-Si (b and e), and a-Si/Ag/a-Si (c and f) electrodes before (a–c) and after (d–f) 150 cycles.

Fig. 5 shows the surface morphologies of the samples before and after 150 cycles. The cracking of the Ag/a-Si electrode is more fundamental than that of the a-Si/Ag/a-Si electrode, which can be the reason for the Ag/a-Si electrode possessing smaller areal capacity retention after 150 cycles. As shown in Fig. 5(d), pure silicon thin film electrode is fractured and even peeled off the copper foil, indicating the worst adhesion between the electrode and the copper current collector compared with that observed for the Ag/a-Si and a-Si/Ag/a-Si electrodes. The amount of remaining Si coincides qualitatively with the cycling properties.^58^ This suggests that the addition of a silver thin film deposited on top of (Ag/a-Si) or incorporated between the amorphous Si thin film layers (a-Si/Ag/a-Si) improves the structural stability to survive the drastic volume change and the huge mechanical stress during cycling.

**Fig. 6:**
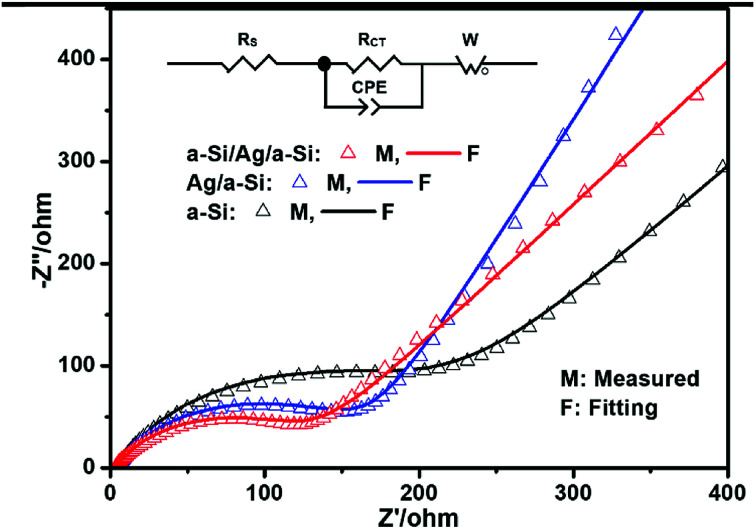
Electrochemical impedance spectra of pure a-Si, Ag/a-Si, a-Si/Ag/a-Si electrodes after 5 cycles.

The cycling performances of pure a-Si, Ag/a-Si, and a-Si/Ag/a-Si thin film electrodes are further studied by electrochemical impedance spectroscopy (EIS) measurements (Fig. 6) after 5 cycles. *R*_s_ is the ohmic series resistance associated with the cell components such as the electrolyte or other components,^59^ and *R*_CT_ is the charge transfer resistance corresponding to the diameter of the semicircle at high frequency; *W* is the Warburg impedance related to the Li ion diffusion into the active material, which is generally identified by a sloping straight line. The capacitor components are replaced by constant phase elements (CPE).^11,46^ The diameters of the semicircle for the Ag/a-Si (*R*_CT_, 156 Ω) and a-Si/Ag/a-Si (*R*_CT_, 115 Ω) electrodes are significantly smaller than that of the pure Si (*R*_CT_, 178 Ω) electrode, revealing lower charge-transfer impedances. It is indicated that the introduction of a silver film can enhance the conductivity of a silicon thin film. It is also recognised that Si-based electrodes generally exhibit cracking and crumbling during lithium insertion and extraction. After the recycling process, partial broken silver films in Ag/a-Si and a-Si/Ag/a-Si electrodes, converted into fillers form and reinserted these cracks. This restructured morphology, as a conductive percolation network, enhanced the cycling performance of Si-based electrodes. Therefore, the silver thin film located on the surface of or in the middle of the silicon film can enhance the stability of Si-based electrodes during the charge/discharge processes. In our study, the Ag film as an embedded layer plays two main roles, especially in the multilayer a-Si/Ag/a-Si structure. On the one hand, the Ag layer is ductile enough to relieve the stress caused by large volume changes owing to Li alloying and dealloying reactions with Si. On the other hand, Ag has attracted great attention due to its excellent electric conductivity and its negligible effects on the lithium storage behavior of Si.^57^

**Fig. 7:**
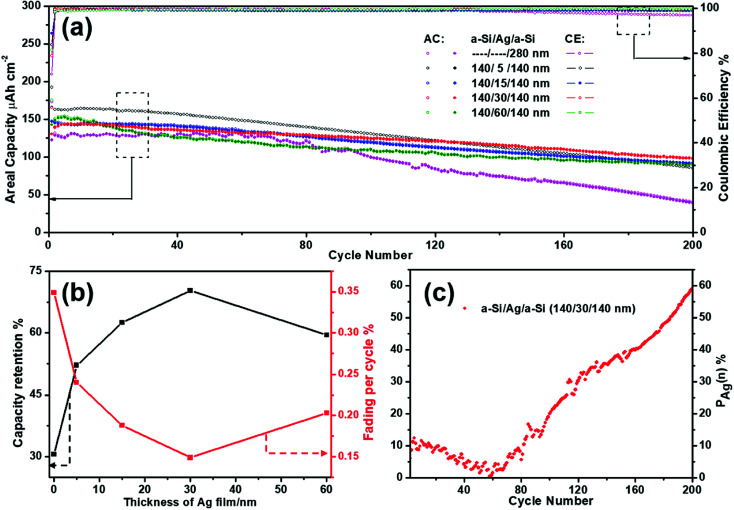
(a) The cycling performances of multilayer a-Si/Ag/a-Si electrodes with different layer thicknesses of silver (5, 15, 30, and 60 nm) and pure Si electrode are compared at a current density of 65.2 μA cm^−2^ within the voltage range of 0.01–1.5 V. The solid and hollow symbols correspond to the charge and discharge curves. “AC” and “CE” stand for “Areal Capacity” and “Coulombic Efficiency”, respectively. (b) Reversible capacity retention and fading per cycle values with different thicknesses of silver thin films. (c) Capacity proportions contributed by the silver thin film based on a-Si/Ag/a-Si (140/30/140 nm) electrode during 2nd to 200th cycles.

It has been mentioned above that silver thin film layers incorporated between the amorphous Si thin film layers (a-Si/Ag/a-Si) can enhance the cycling performance of the silicon thin film electrode. In this section, the a-Si/Ag/a-Si electrode was selected to study the optimum thickness of the Ag layer as well as the influence of the Ag layers on the electrochemical performance. The cycling performances of the a-Si/Ag/a-Si electrodes with different thicknesses of silver layers (5, 15, 30, and 60 nm) are compared. For better comparability, the cycling data of a pure amorphous Si thin film (280 nm) are also depicted in Fig. 7(a). All electrodes containing silver as an additional interlayer show superior cycling performances from the 1^st^ to the 200^th^ cycle compared to a pure Si thin film. The initial reversible discharge areal capacities are 162.8, 146.4, 139.6, and 150.6 μA h cm^−2^ and the discharge areal capacities decay to 84.9, 91.6, 98.1, and 89.7 μA h cm^−2^ after 200 cycles, indicating the capacity retentions of 52.2%, 62.6%, 70.3%, and 59.6% for 5 nm Ag, 15 nm Ag, 30 nm Ag, and 60 nm Ag, respectively (Fig. 7(b)). The pure Si electrode displays the lowest capacity retention value at only 30.6% after 200 cycles. The electrode with 30 nm Ag between two layers of Si shows the highest capacity retention during charge/discharge cycling and the lowest decay rate (0.15%) per cycle.

The contribution of the silver thin film is mainly studied based on the a-Si/Ag/a-Si (140/30/140 nm) electrode. The capacities contributed by the Ag thin film electrode can be approximately obtained by carrying out the subtraction calculation^41^ in the corresponding cycles by referring to the following equation:

1 *C*_Ag_(*n*) = *C*_a-Si/Ag/a-Si_(*n*) − *C*_a-Si_(*n*)

Here, *n* is the cycle number from 2 to 200; *C*_a-Si/Ag/a-Si_(*n*) and *C*_a-Si_(*n*) stand for the discharge capacities of a-Si/Ag/a-Si and pure a-Si electrodes, respectively, in the corresponding cycle. For the a-Si/Ag/a-Si electrode, *C*_Ag_ retains a value of more than 58 μA h cm^−2^ even after 200 cycles. To comprehend the contribution to the total capacity, the proportion contributed by the silver thin film layer during cycling is given by the following equation:

2 *P*_Ag_(*n*) = *C*_Ag_(*n*)/*C*_a-Si/Ag/a-Si_(*n*) × 100%

Fig. 7(c) reveals the positive effect of silver thin film after continuous cycling when incorporated between amorphous Si thin film layers. It can be seen that *P*_Ag_(*n*) shows a slight declining trend before 60 cycles; then, it increases notably up to 59.1% in the remainder of the 200 cycles. The above variation in tendency is due to the interaction of a-Si/Ag/a-Si and pure a-Si thin film electrode cycles. The intercalated a-Si/Ag/a-Si structure is equivalent to reducing the thickness of a single a-Si film layer by forming two a-Si film layers with the same thickness of 140 nm. Previous research^13,21,58^ results show that a thinner a-Si film layer exhibits more stable cycling performance and the protective behavior of a silver film also improves the electrochemical kinetics by restraining the volume expansion to avoid mechanical breakage.

**Fig. 8:**
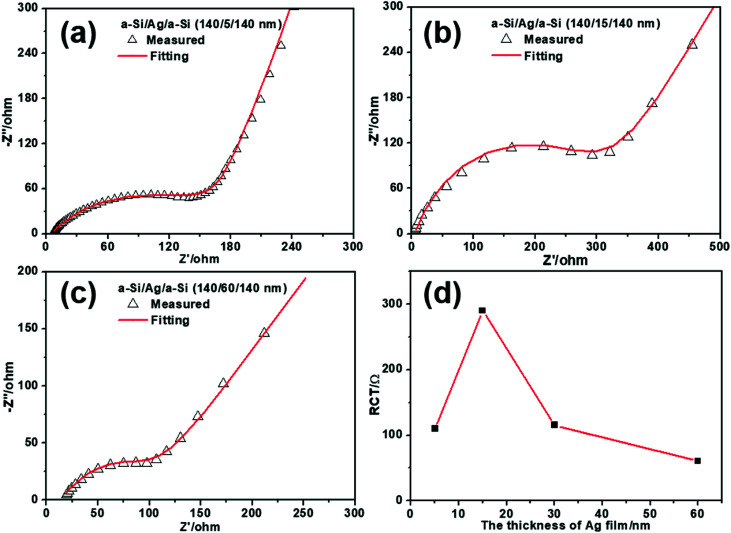
Electrochemical impedance spectra of multilayer a-Si/Ag/a-Si electrodes with different silver layer thickness of 5 nm (a), 15 nm (b), and 60 nm (c) after 5 cycles. (d) The calculated charge transfer resistance (*R*_CT_) results of the multilayer a-Si/Ag/a-Si electrodes with different layer thicknesses of silver.

To further study the mechanism of Li^+^/electron transport in multilayer a-Si/Ag/a-Si electrodes, electrochemical impedance spectra (EIS) of a-Si/Ag/a-Si electrodes with different layer thicknesses of silver (5, 15, 30, and 60 nm) after the first five cycles were obtained, as shown in Fig. 8(a), (b), 6 and 8(c), respectively. The equivalent circuit of the EIS impedance simulation is similar to that shown in Fig. 6. It is reported that the *R*_CT_ value decreased along with increasing silver content because the silver nanoparticles provided a favorable environment for the migration of lithium ions into/from the silicon particles.^11^ However, this is not the case for the multilayer structured electrodes. In our study, the *R*_CT_ values of the a-Si/Ag/a-Si electrodes measured with different layer thicknesses of silver (5, 15, 30, and 60 nm) are 110, 290, 118, and 60.5 Ω, respectively, as shown in Fig. 8(d). It is suggested that the *R*_CT_ values of the multilayer a-Si/Ag/a-Si electrodes decrease with an increase in the thickness of the silver layer except for the silver layer thickness of 5 nm. The above results are explained by the interaction of silver content, the mutual mixing degree and the crack size of the Si-based film during the lithium insertion/extraction processes and the mechanism is explained below.

**Fig. 9:**
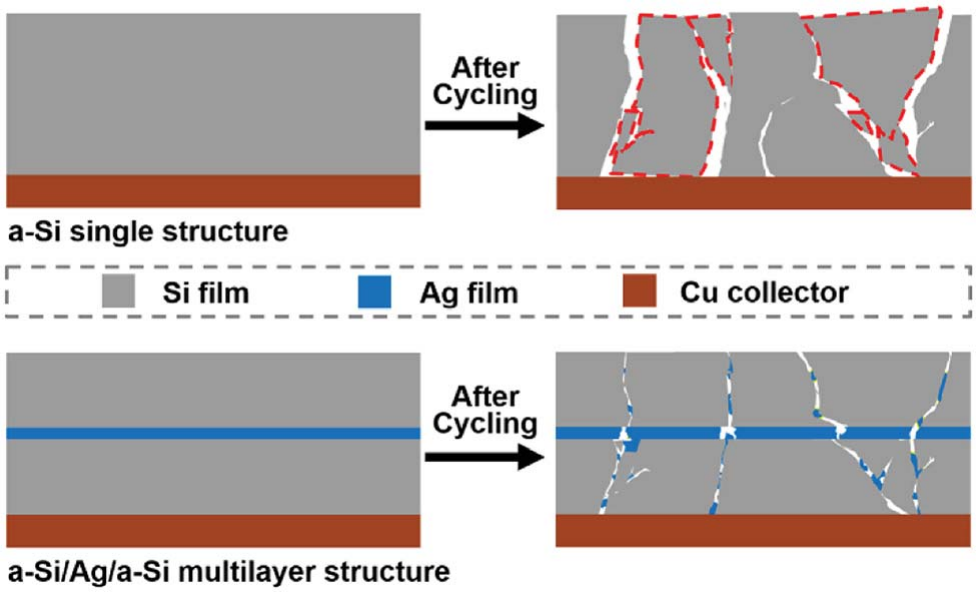
Schematic illustration of the mechanism of an a-Si/Ag/a-Si electrode for improving the electrochemical performance. The areas marked by red dashed lines represent the inactive silicon materials after cycling. After cleaning, the inactive silicon materials are removed and the surface of the copper foil will be exposed.

On the one hand, increasing the silver content can effectively enhance the electron conductivity of the silicon-based electrode.^11^ When the silver content is abundant, other factors play a minor role. Thus, the value of *R*_CT_ tends to decrease when the thickness of the silver layer increases from 15 to 60 nm. On the other hand, silicon-based multilayered anode materials will intermix after cycling,^29^ which also leads to lower *R*_CT_. Because of the severe volume change, Si-based film electrodes tend to crack and pulverize during cycling. Meanwhile, a part of the broken silver layer or particles is converted to fillers and enters the cracks, which results in a conductive percolation network in the Si-based electrodes, as shown in Fig. 9. As a result, the silver fillers and the broken silicon have undergone a certain degree of mixing. At this time, the level of reciprocal mixing can also affect the magnitude of the charge transfer resistance. When the thickness of the silver film and the size of the cracks reach a certain degree, they favor intermixing, and the degree of intermixing plays the most important role. Therefore, it is reasonable that the a-Si/Ag/a-Si electrodes with silver layer thickness of 5 nm exhibit a lower *R*_CT_ value than the a-Si/Ag/a-Si electrodes with silver layer thickness of 15 nm.

From the observations discussed above, we can confirm that the multilayer a-Si/Ag/a-Si electrode exhibits very excellent electrochemical performance compared to the pure silicon film electrode. This positive influence can be ascribed to two reasons: first, the additional interlayer of ductile silver acts as a buffer layer as it is a compact layer and experiences only slight volume expansion, which leads to smaller crack sizes after cycling. Second, the broken silver layer acts as a conductive agent between the cracks of the silicon film. Silver nanosheets can connect the broken inactive silicon film portions together and convert them back into an active substance. Therefore, the multilayer a-Si/Ag/a-Si electrode has higher areal capacity after 200 cycles and the charge transfer resistance decreases along with increasing silver content.

## 4. Conclusions

In this study, Ag/a-Si, a-Si/Ag/a-Si, and a-Si/Ag thin film layer electrodes carefully designed and fabricated by the magnetron sputtering method to stabilize the cycling performance of the silicon thin film layer electrodes were thoroughly investigated. The results show that the silver thin film incorporated between the amorphous Si thin film layers (a-Si/Ag/a-Si) not only displays high and stable capability (with the highest discharge areal capacity of 111.7 μA h cm^−2^ and optimal capacity retention of 80% after 150 cycles at 65.2 μA cm^−2^ compared to other electrodes), but also exhibits the best rate performance compared to other electrodes. It also possesses lower charge-transfer impedances due to excellent electron conductivity of silver. The structure of the a-Si/Ag film as the electrode material shows the worst cycling performance, which indicates that the silver thin film deposited on the copper foil cannot increase the adhesion of silicon to the substrate. The Ag thin film layer deposited on the surface of an a-Si layer (Ag/a-Si) significantly improves the electrochemical performance in terms of an enhanced coulombic efficiency because the surface layer of silver can effectively protect the highly reactive Si surface from the bulk electrolyte. In these studies, the influence of the Ag thin film layer thickness was also examined. With the increase in the thickness of silver film, the capacity retention increases first and then decreases. A critical thickness of 30 nm is found for the silver thin film layer, at which the highest capacity retention (70% after 200 cycles) can be achieved. This study provides a way to improve the electrochemical performance of a silicon thin film layer electrode with an additional silver layer as the interlayer (a-Si/Ag/a-Si).

## Conflicts of interest

There are no conflicts to declare.

## Supplementary Material
